# A Scoping Review and Population Study Regarding Prevalence and Histopathology of Juvenile Vulvar Melanocytic Lesions. A Recommendation

**DOI:** 10.1016/j.xjidi.2022.100140

**Published:** 2022-06-22

**Authors:** Beth Morrel, Irene A.M. van der Avoort, Jeffrey Damman, Antien L. Mooyaart, Suzanne G.M.A. Pasmans

**Affiliations:** 1 Department of Obstetrics and Gynecology, Erasmus MC University Medical Center Rotterdam, Rotterdam, The Netherlands; 2 Department of Dermatology, Center of Pediatric Dermatology, Sophia Children’s Hospital, Erasmus MC University Medical Center Rotterdam, Rotterdam, The Netherlands; 3 Department of Obstetrics and Gynecology, Ikazia Hospital, Rotterdam, The Netherlands; 4 Department of Pathology, Erasmus MC University Medical Center Rotterdam, Rotterdam, The Netherlands

**Keywords:** AGN, atypical genital nevus, JVLS, juvenile vulvar lichen sclerosus, LS, lichen sclerosus, MVM, malignant vulvar melanoma, VLS, vulvar lichen sclerosus

## Abstract

Cases of vulvar melanocytic lesions in juveniles are rarely reported. We analyze the evidence regarding vulvar melanocytic lesions in juveniles with or without vulvar lichen sclerosus to help decision making by clinicians and pathologists. A scoping review on vulvar melanocytic lesions with or without vulvar lichen sclerosus, including malignant vulvar melanomas, in females up to age 18 years was performed. In addition, the histopathology records of the cohort of all such lesions in The Netherlands from 1991 through 2020 were investigated, and a structured analysis of tissue samples of the subset of cases with lichen sclerosus was performed. The literature study performed confirms that vulvar melanomas in juveniles are extremely rare and that published case reports are often disputed. In The Netherlands, there are no cases of malignant vulvar melanomas up to age 18 years recorded from 1991 through 2020. Atypical histopathological features are often found in biopsies of vulvar nevi in juveniles, especially with concomitant lichen sclerosus, confirming earlier case studies in the literature. We conclude that even with atypical findings, vulvar melanocytic lesions in juveniles have a benign course. To avoid unnecessary and possibly mutilating procedures, we advise referral to an expert center and adaption of existing guidelines for vulvar melanocytic lesions in juveniles.

## Introduction

Vulvar melanocytic lesions in the background of lichen sclerosus (LS) are an enigma for both clinicians and pathologists, especially when found in a juvenile (a child or adolescent up to age 18 years). The nevus may first be noted when the child is seen because of complaints due to vulvar LS (VLS), and the lesion may be damaged by scratching. In certain locations such as genitalia and acra, melanocytic lesions are considered special-site nevi and may show histopathological signs of atypia, although having a benign course (Clark et al., 1998).

It is estimated that 10% of adult women have a pigmented vulvar lesion of some kind, including melanosis, lentigo, and nevi (Hunt et al., 2014; Murzaku et al., 2014), and approximately 2.3% have a vulvar nevus (Rock et al., 1990). Prevalence in juveniles is uncertain (Hunt et al., 2014; Trager, 2004).

Of the case reports of malignant vulvar melanomas (MVMs) in juveniles, most were found in the background of LS (La Spina et al., 2016). A number of these diagnoses were challenged (Carlson et al., 1997; Schaffer and Orlow, 2005). Clinicians are encouraged to be liberal with biopsies of pigmented vulvar lesions in adult women to rule out melanoma and expedite early detection (Heller, 2013). Regarding children, there is no such directive (Trager, 2004). Pathologists generally advise a diagnostic excision if there are atypical features in a biopsy of a vulvar melanocytic tumor. Such a directive for juveniles might lead to unnecessary and possibly mutilating excisions.

Which specific aspects of melanocytic lesions of the vulva in juveniles should clinicians and pathologists be aware of? What is the role, if any, of concomitant LS? What is the prevalence of MVM in juveniles?

We examine the risk of vulvar melanocytic lesions in a juvenile being or becoming malignant by performing a literature study together with a nationwide population study. The histopathological characteristics of vulvar melanocytic lesions found in the literature are then applied to a case series of the national cohort with concomitant LS. We discuss the need for specific guidelines for the care of juveniles with vulvar melanocytic lesions.

## Results

### Scoping review of the literature

**Figure 1:**
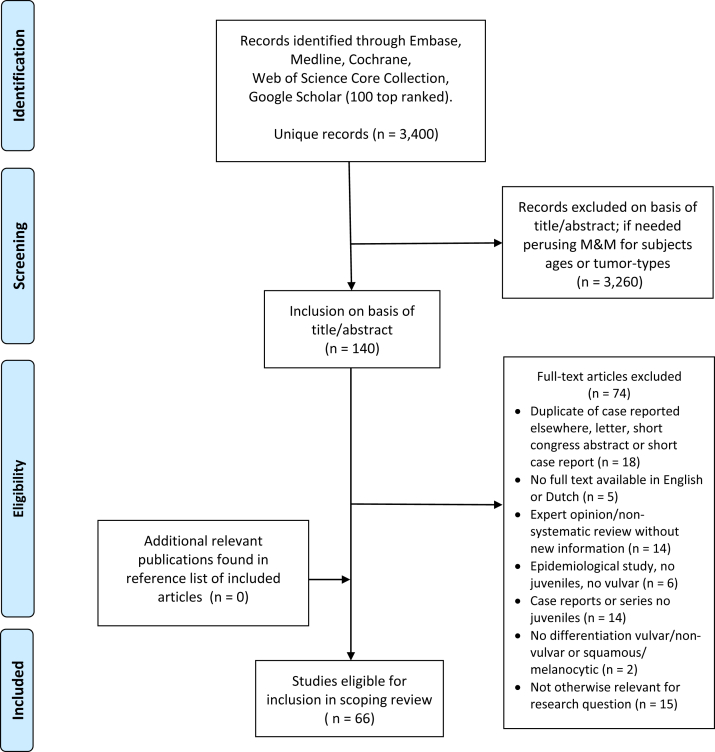
**Flowchart of inclusions and exclusions in the scoping review of vulvar melanocytic lesions in juveniles.** M&M, material and methods of the publication.

The literature search yielded 3,400 publications, with 140 eligible after screening. On the basis of full texts, 66 publications were included, as shown in Figure 1 and summarized in Table 1.

**Table 1:** Summary of the Literature Found in a Scoping Review of Vulvar Melanocytic Lesions in Juveniles

Reference	Subject of Article	Type of Article	Number of Cases and age, y, if known	LS, Yes/No (Considered)	Results and Remarks
**Case report of juvenile vulvar melanoma**					
Egan et al., 1997	Childhood vulvar melanoma	Case report	2 cases, ages 9 and 11	Yes	Show the clinical and histological findings in two cases with VLS; shave biopsies and subsequent excisions showed AGN, with no residual melanoma. For discussion on whether these two cases are truly malignant melanomas, see Carlson and Mihm (1997) and Egan et al., (1997)
Filippetti and Pitocco, 2015	Childhood vulvar melanoma	Case report	1 case, age 14	No	Amelanotic vulvar melanoma, discuss rarity of this lesion
Friedman et al., 1984	Childhood vulvar melanoma	Case report	1 case, age 14	Yes	Discuss that vulva melanoma in general is aggressive. This case has a favorable outcome; no positive nodes, diagnosed as superficial spreading melanoma
Hassanein et al., 2004	Childhood vulvar melanoma	Case report	1 case, age 10	Yes	Lengthy description of histology, leads to discussion of validity of diagnosis; see the letter of Schaffer and Orlow (2005) refuting diagnosis and authors' reply Hassanein and Wilkinson (2005).
Hulagu and Erez, 1973	Childhood vulvar melanoma	Case report	1 case, age 9	n.s.	Case report clitoral melanoma, local excision only; no positive nodes, favorable outcome
La Spina et al. (2016)	Vulvar melanoma and its association with VLS in a child	Case report and review	1 case, age 11	Yes	Vulvar melanoma is rare and differs from cutaneous melanoma: 1 in 10 of general population has a pigmented vulvar lesion; discuss relation melanoma and VLS; at this publication, 10 cases of MVM with LS in literature, five children, five adults; differential diagnosis between benign nevus and melanoma in the setting of VLS could be difficult; markers discussed; description of histology of nevus versus melanoma in VLS (in children); possible markers include KIT, PDGFRA, HMB-45, and Ki-67.
Rosamilia et al., 2006	Childhood vulvar melanoma	Case report	1 case, age 10	yes	This is the case in the series of Wechter et al. (2004; two locations with one positive lymph node; two melanomas with positive node, lymphadenectomy, and interferon; NED after 32 months; in their previous report Wechter et al. (2004), VLS was not mentioned; this is their rectification.
Webber et al., 2008	Adolescent vulvar melanoma	Case report	1 case, age 12	n.s.	Describe histopathology; congress abstract not subsequently published
**Case report of juvenile vulvar nevus**					
Bussen, 2009	Junctional melanocytic nevus of the vulva with LS	Case report	1 case, age 9	Yes	Show diagnostic difficulty of vulvar melanocytic nevus in LS in child mimicking melanoma
Hoffmann et al., 2011	Perianal nevus in juvenile girl	Case report	1 case, age 12	No	Describe the similarities and differences with AGN of the vulva in girls, histological description, no concomitant LS, 10-year follow-up
Lebeau et al., 2006	Vulvar pemphigoid in a child	Case report	1 case, age 8	No	A nevus in background of an autoimmune disease; shows atypia and basal proliferation in an AGN
Mandal et al., 2009	Acquired clitoromegaly due to a nevus in a child	Case report	1 case, age 8	No	Description of case, differential diagnosis of clitoromegaly
Mulcahy et al., 2013	Nevus with LS	Case report in letter	1 case, age 7	Yes	Clinicopathology and histopathology information; discussion of relationship with VLS apply HMB-45
Neri et al., 2016	Nevus with LS	Case report and review	1 case, age 8	Yes	Immunohistochemistry used HMB-45, MART-1, and p16
Pinto et al., 2012	Genital melanocytic nevi in juveniles	Case report and review	1 case, age 7	Yes	Discuss possible role of LS in malignant transformation of melanocytes analog to risk of VSCC in the background of LS; there can be a pseudomalignant melanocytic phenomena in inflammatory conditions; look at histological features and macroscopic (clinical) features; literature of all the five cases of MVM in child in the background of (V)LS and seven cases described as AGN in the background of genital LS; staining used melan-A and Ki-67; conclude in retrospect that this is an AGN, not a malignant melanoma
Polat et al., 2009	Spitz nevus of the vulva	Case report	1 case, aged 11	No	First published vulvar Spitz nevus, discuss differential diagnosis with melanoma
Rotunno et al., 2019	Disseminated spitzoid nevi in child, including vulvar lesions	Case report	1 case, age 6	No	A case description stating that there are a few case reports
Schaffer and Orlow, 2005	Reaction to article by Hassanein et al. (2004)	Case report, letter	1 case, age 8	Yes	Reference both Carlson et al. (2002, 1997) and Clark et al. (1988) that 1 of 3 genital nevi is misdiagnosed and that diagnosis is even more difficult in the setting of LS; state that activated melanocytic phenotype related to cytokine milieu and altered extracellular matrix in LS; bridge a number of cases. Egan et al., 1997, Egan and Vanderhooft, 1997, Friedman et al. (1984), Hassanein et al. (2004), and Hassanein and Wilkinson (2005), concurring with Clark et al. (1998); refute diagnosis in article by Hassanein et al. (2004) and discuss a case.
Spatz et al. (1998)	Vulvar blue nevus	Case report	1 case, age 12	No	Case report of malignant vulvar blue nevus: nodule seen at age 12 years, malignant blue nevus at age 28 years, and malignant ovarian metastasis at age 43 years, 15 years after removal and diagnosis of malignant blue nevus
Tan et al., 2020	Balloon cell vulvar nevus, a melanocytic lesion	Case report	1 case, age 15	No	Mild cellular and nuclear atypia seen; discuss the diagnosis of balloon cell nevus
Trager, 2004	(Congenital) vulvar nevus	Case report and review	1 case, age 3	No	Rarity of MVM, give advice on how to follow congenital nevus
Yamazhan et al., 2012	Vulvar cellular blue nevus in adolescent	Case report	1 case, age 15	No	Description of case and differential diagnosis
Zhou and Crowson, 2003	Milk line nevus	Case report	1 case, age 17	No	Describe histological features of atypical milk line nevus with; discuss phenomenon of Pagetoid spread and nesting
**Case serie nonmelanomas**					
Carlson et al., 1997	Differential diagnosis melanoma and nevi	Letter	n.r.	n.r.	Refute diagnosis of melanoma in cases of Egan et al. (1997)
Carlson et al., 2002	Differential diagnosis LS, melanocytic nevi and MVM	Case series	11 cases/4 juveniles	Yes	In a series comparison of persistent nevi, persistent melanoma, compound nevi; HMB-45 is more intense with LS and does not differentiate for melanoma; Ki-67 expression is higher in malignant melanoma.
El Shabrawi-Caelen et al., 2004	Genital pigmented lesions and LS	Case series	5 cases/4 males, 1 female, age 6	Yes	Description of histology; HMB-45 in activated melanocytic phenotype; address diagnostic challenge of these lesions; discuss post-inflammatory pigmentary alterations as explanation for hyperpigmentation in LS; genital melanocytic nevi are also diagnostic challenge
Gleason et al., 2008	Clinical and morphological features of cases & review	Case series	55 females, 22 cases aged ≤20	Yes	Historical series; 80% moderate-to-severe cellular atypia; most important differential diagnoses are DN and melanoma; only one subject with a history of VLS; says association of VLS with AGN is rare and does not concur in our data.
Hunt et al., 2014	Childhood genital nevi	Case series	40 cases, 17 females	Yes	Retrospective chart review of over 10 years of practice; 3.5% of pediatric nevi were genital (40 of 1,159), male: female ratio is 1.3:1; no genital melanoma, one dysplastic nevus in background of VLS; two cases with atypia; 63% seen before age 2 years, cases seen in 11-year period; mean follow-up of 1.5 years
Michalova et al., 2017	DSIL lesions overlying melanocytic nevi	Case series	30 cases, age 4‒68; 8 females aged ≤19	n.s.	Coin the term DSIL, young group of patients; markers p53 and melanocytic markers s-100, SOX-10, melan A, and cytokeratin AE1/3 to differentiate melanocytes and keratinocytes; only one (adult) case was associated with VLS; other statistics than our cohort
Ribé, 2008	AMNGT as a distinct entity in young women	Case series	58 genital nevi, 6 cases of AMNGT	n.s.	Conclude that AMNGTs are not precursors to dysplastic nevi or melanoma; mean age of cases of AMNGT is 21 years; atypical/dysplastic in younger subjects, no mention of VLS
**Case serie melanomas**					
Ariel, 1981	MVM	Case series and review	45/1 juvenile, age 15	n.s.	Distribution of melanoma in female genital tract and 5-year survival (literature); the one juvenile (aged 15 years) was seen before 1965. No further description of the case. In the course of 45 years, possibly one vulvar melanoma was recorded; 1981.
Chung et al., 1975	Role of depth of invasion in MVM	Case series	44/1 juvenile, age 17	n.s.	Correlating depth of invasion to survival; specifics juveniles not given
De Simone et al., 2008	Mvm	Case series	10 cases/ 1 juvenile, age 15	n.s.	All other cases were aged ≥50 years, the juvenile had an in-situ superficial spreading tumor.
Eleno Beierbach et al., 2020	Pediatric melanoma, genital/non-genital	Case series	16 pediatric melanoma, 1 vulvar	n.s.	The outcome of melanomas in pediatric practice; sentinel node done; outcome not differentiated; pathology not verified in article.
Jaramillo et al., 1985	MVM survival	Case series	16 cases, age 18‒89, 3 cases aged ≤40 not further specified	n.s.	Discuss pelvic lymphadenectomy; treatment and clinical course juvenile not stated
Morgan et al., 1988	MVM clinical description and role of therapy	Case series	18 cases, 1 juvenile, age 18	n.s.	Treatment option based on the level of disease (using historical grading of tumor level); the patient aged 18 years with level II disease treated with wide local excision, NED after 7 years; the adolescent was the only subject in series where no groin nodes were excised
Nagarajan et al., 2017	MVM descriptive statistics prognosis various factors	Case series	100 cases, 1 juvenile, age 18	n.s.	Prognostic value: tumor thickness and tumor mitotic rate; propose a new classification of tumor thickness; specifics of juvenile case not given.
Räber et al., 1996	MVM prognostic value various factors, including age	Case series	89 cases, 1 juvenile, age 18, mean age of 59.4	n.s.	Biologic similarities between genital and extragenital melanoma, primary surgery important, base clinical management on depth of invasion and ulceration; retrospective analyses of prognostic value of age, Breslow thickness, Clark level of invasion, positive nodes, site, postoperative staging; all cases from three hospitals; information regarding one juvenile not given, thus one case of vulvar melanoma in juvenile in population study from three hospitals in Germany during 13 years.
Rao et al., 1990	Melanoma in children, outcomes	Case series	33 cases, one perineum in juvenile, age 8	n.s.	Survival closely correlated with stage of disease; the juvenile MVM had very low Breslow thickness compared with those of most other cases.
Rouzbahman et al., 2015	Vulva and vaginal melanoma, histopathology and genetic mutations	Case series	44 cases, 33 vulvar, 1 juvenile, age 17	n.s.	Case series from Toronto Canada looking at genetic markers *BRAF*, *c-KIT*, *NRAS* mutations in vulvar melanomas; single center, probably same population as Sinasac et al. (2019).
Sinasac et al., 2019	Diagnosis and outcome in a case series of vulva and vaginal melanoma	Case series	68 cases, 50 vulvar, 1 juvenile, age 17		Cases over 12 years seen in referral center in area of Toronto Canada; aside from stating age, no differentiation for the results of the adolescent, no mention of LS; no clinical information, possible overlap with population Rouzbahman et al. (2015).
Trimble et al., 1992	MVM age, staging, and survival	Case series	80, probably 2 juveniles	n.s.	Younger age prognosis best; not clear about how many juveniles and their specific survival; overlap with population reported by Chung et al. (1975).
Wechter et al., 2004	MVM comparing results on location, symptoms, and outcome to literature	Case series	20, age range of 10‒93. One juvenile, age 10, all others aged ≥37	n.s.	Literature results show positive lymph node as most powerful predictor of poorer survival; only juvenile case ever reported with positive sentinel node; see Rosamilia et al. (2006); the patient aged 10 yeas was documented as having two primary melanomas and one positive groin node ipsilateral.
**Cohort studies**					
Hieta et al., 2019	Association LS and MVM	Cohort, brief communication	9 MVM, age not given	Yes	What is association of LS and vulvar melanoma: LS gives relative risk of melanoma of 341; population study search (Finland); LS and melanoma 2000‒2013
Rock et al., 1990	Prevalence of vulvar nevi in general practice	Cohort	301 consecutive women, 1 juvenile, aged 19	n.s.	Ask prevalence of vulvar pigmented lesions and nevi, lentigo at somewhat higher age; 2.3% had vulvar nevus, the patient aged 19 years was the only case of dysplastic nevus; gives frame of reference, percentage of female population with vulvar nevi
Woolcott et al., 1988	Mvm Australia 1955-1987	Cohort	50 cases, range 15‒91, at least one juvenile	n.s.	Information found through all oncology centers, prognosis in all therapy groups survival <50%; prognosis as related to age not discussed, no description of histology
**Epidemiology**					
Blessing et al., 1991	Scotland (1979–1989)	Epidemiology	41, range of 11‒92, and 37 of the cases aged ≥50, 1 case aged 11, 3 cases aged 40‒50.	n.s.	Vulva melanoma was 1.7% of all vulvar melanomas in females, poor survival; Age gap between the one juvenile and the rest, no information about the individual case
Cohen Goldemberg et al., 2020	Prevalence of mucosal melanoma in Brazil all sites 2000-2016	Epidemiology	801 mucosal melanomas, 270 vulva‒vagina‒cervix, of which three cases aged 10‒19	n.s.	Prevalence per location,3 cases documented from Brazil of adolescent MVM
Ragnarsson-Olding et al., 1993	Vulva melanoma in Sweden	Epidemiology	245 cases involving vulva and vagina, 4 cases aged 15‒29 not further specified. Range of 18‒91, overall mean age of 67.7	n.s.	Age distribution, survival; probably only one case of juvenile vulvar melanoma in the 25-year period studied
Sanchez et al., 2016	Population study of genitourinary melanomas in men and women in United States: 1973‒2010, data from SEER 1973‒2010	Epidemiology	1,568 cases, 1,463 (93%) women with vulvar‒vaginal melanoma, 13 females aged 10‒19, and 64 females aged 20‒29	n.s.	Location, age, sex, survival; 100% 10-year survival in cohort aged 10‒19 years; 75.3% of cases involving vulvar; vaginal melanoma with poorer survival; SEER data possible overlap with several of the case reports
**Histopathology and markers**					
Ahn et al., 2016	Anatomical regions with known site-related atypia	Narrative, educational	0	Yes	Benign nevi mimic dysplasia or melanoma in specific sites owing to microanatomy; refer to and synthesize Gleason et al. (2008), Clark et al. (1998), and Carlson et al. (2002), stating “across almost all lesions, a prominent feature of nevi of genitalia is marked asymmetry, junctional proliferation of round-to-oval nests with striking areas of confluence as well as single melanocytes at different levels of the epidermis, including the stratum corneum. In some cases, the entire basal layer can be replaced by single melanocytes. In the nested pattern, oval nests are often oriented either perpendicularly or parallel to dermoepithelial junction.”
Blessing, 1999	Melanocytic nevus with atypical junctional activity	Narrative	0	n.s.	Natural history of acquired common nevus; discuss various types of nevus; Spitz nevus: all stages can mimic melanoma; Pagetoid Spitz nevus to be distinguished from melanoma; Spindle cell nevus; Halo nevus; recurrent and traumatized nevus; UVR and (acral) melanocytic nevus; genital nevus: especially in premenopausal women; assess classification and proposes strategy
Brenn, 2011	AGN vs MVM	Review	0	Yes	Overview AGN versus MVM; clinical and histological features of AGN with distinction from vulvar melanoma; primarily histological review; refers to Clark et al. (1998)
Brenn, 2018	Differentiating various nevi from melanoma	Narrative	0	n.s.	Systematic description of differences in various types of nevi from melanoma
Christensen et al., 1987	Comparison of histology of vulvar nevi to non-vulvar nevi	Case series/case controlled	57 vulvar compared (2 aged ≤19) with 200 torso nevi (all aged ≥20)	No	No evidence found for increased risk of precursors to melanoma in vulvar nevi
Clark et al., 1998	Histopathology of AMNGT, MM, DN	Case series	56, of which 36 are AGN	No	Revisions of diagnosed melanocytic lesions, MVM genital, seminal publication, gives criteria to use when interpreting findings, basis for discussion on most subsequent publications; 36 are atypical melanocytic nevus of the genital type; describes three types of MVM and give guidelines for clinical management; with revision, many lesions were not confirmed to be MVM; 30% of AMNGT initially misdiagnosed as melanoma
Cook, 2010	Special site nevi	Mini symposium	0		NOSS description per anatomical site
Elder, 2006	Nevi of special sites	Narrative	0	No	Describe melanoma versus dysplastic nevus versus atypical nevus of special site; educational
Fu et al., 2021	Density of melanocytes in VLS	Case control	30 cases, 15 controls, 7 early, and 8 late VLS	Yes	Density and thickness of epidermis in VLS, found fewer melanocytes in VLS
Haupt and Stern, 1995	Pagetoid melanocytosis in different types of melanocytic lesions	Case series	218 melanocytic lesions, 5 of the vulva (ages 22‒31)	No	Included because of histological information, discuss in which nevi is there Pagetoid melanocytosis: in vulva nevi, 80%, highest rate except for melanomas; describe Pagetoid melanocytosis: upward discontinuous extension of melanocytes into the superficial epidermis
Mason et al., 2011	Overview and examples of NOSS	Review	0	No	Conclude no clear diagnostic criteria for NOSS, which gives pitfall for over diagnosis of melanoma; coin the abbreviation NOSS = nevus of special site; well-written, systematic examples of different patterns in NOSS
Skelton et al., 1991	HMB-45 staining in melanocytic lesions	Case series	225, 30 from hormonal-reactive areas not otherwise specified	No	HMB-45 is positive in the majority of various melanocytic lesions, not in commonly acquired nevi
Wick, 2015	Benign lesions that may be confused with melanoma	Narrative	0 vulva	No	Illustrates various types of nevi with aspects of histopathology; applicable to genital nevi and MVM
**Otherwise relevant**					
Gadducci et al., 2018	Female genital malignant melanoma	Systematic review	0	Yes	Comprehensive information on all aspects of genital melanoma; surgery treatment of choice; systematic review of epidemiology
Murzaku et al., 2014	Vulvar melanocytic lesions	Review and narrative	0	Yes	Clinical and histopathological features of vulvar melanocytic lesions, flow diagram for clinicians to distinguish vulvar nevi, melanosis, and melanoma from each other
Strickland and Fadare, 2021	Pediatric vulvar malignancies	Systematic review	100	Yes	Literature in 1970‒2020, age ≤21 years. A total of 100 cases of vulvar malignancy found, 50% rhabdomyosarcoma, 6 MVM.

Abbreviations: AGN, atypical genital nevus; AMNGT, atypical melanocytic nevus of the genital type; DN, dysplastic nevus; DSIL, differentiated squamous intraepithelial lesion; LS, lichen sclerosus; MVM, malignant vulvar melanoma; NED, no evidence of disease; NOSS, nevus of special site; n.r., not relevant; n.s., not stated; SEER, surveillence, epidemiology and end results program; VLS, vulvar lichen sclerosus; VSCC, vulvar squamous cell carcinoma,

#### Case reports of vulvar melanoma in juveniles

We discerned eight publications with case reports of vulvar melanomas in girls aged 9‒18 years encompassing, in all, nine patients (Egan et al., 1997; Filippetti and Pitocco, 2015; Friedman et al., 1984; Hassanein et al., 2004; Hulagu and Erez, 1973; La Spina et al., 2016; Rosamilia et al., 2006; Webber et al., 2008), six with concomitant VLS and three without. No recurrence or metastasis was reported. Three of the diagnoses of MVM in a juvenile with VLS (Egan et al., 1997; Hassanein et al., 2004) were questioned (Carlson et al., 1997; Egan and Vanderhooft, 1997; Hassanein and Wilkinson, 2005; Schaffer and Orlow, 2005). Schaffer and Orlow (2005) also noted the resemblance to the clinicopathological features of LS nevi in the case of an individual aged 14 years published by Friedman et al. (1984), with two vulvar lesions diagnosed as superficial melanomas with concomitant VLS. This child (Friedman et al., 1984) underwent wide excision of both labia minora and superficial inguinal lymph node dissection in which there was no residual MVM found, only VLS. We found only one case (Rosamilia et al., 2006) reporting a positive lymph node in a juvenile. Currently, the consensus is that such a single metastasis of a melanocyte to an adjacent node is not necessarily indicative of malignancy (Mooi, 2014). No publications report recurrence or mortality during follow-up.

#### Case series of MVM including one or more juveniles

A total of 13 case series were identified including at least one juvenile with an MVM (Ariel, 1981; Chung et al., 1975; De Simone et al., 2008; Eleno Beierbach et al., 2020; Jaramillo et al., 1985; Morgan et al., 1988; Nagarajan et al., 2017; Räber et al., 1996; Rao et al., 1990; Rouzbahman et al., 2015; Sinasac et al., 2019; Trimble et al., 1992; Wechter et al., 2004), of which nine included just one juvenile. These series often stemmed from a historical archive. It is not always clear whether these were unique cases because the same databases were used in several publications. An age gap between the one juvenile in a series and the rest of the subjects was often seen (Table 1).

#### Case reports of vulvar nevi in juveniles other than melanomas

We found 15 case reports (Bussen, 2009; Hoffmann et al., 2011; Lebeau et al., 2006; Mandal et al., 2009; Mulcahy et al., 2013; Neri et al., 2016; Pinto et al., 2012; Polat et al., 2009; Rotunno et al., 2019; Schaffer and Orlow, 2005; Spatz et al., 1998; Tan et al., 2020; Trager, 2004; Yamazhan et al., 2012; Zhou and Crowson, 2003) of nonmelanoma vulvar melanocytic lesions in juveniles, ages 6‒18 years, illustrating diagnostic pitfalls, especially with concomitant LS. Pinto et al. (2012) discuss the possible role of VLS in the malignant transformation of melanocytes, analogous to the risk of vulvar squamous cell carcinoma seen with a background of LS. On the other hand, they note that pseudomalignant melanocytic changes are a phenomenon in inflammatory conditions. Single cases of rare types of nevi were reported (Polat et al., 2009; Rotunno et al., 2019; Spatz et al., 1998; Tan et al., 2020; Yamazhan et al., 2012).

#### Case series of vulvar nevi including juveniles illustrating diagnostic pitfalls

There were seven publications describing a series of vulvar nevi (Carlson et al., 2002, 1997; Gleason et al., 2008; Hunt et al., 2014; Michalova et al., 2017; Ribé, 2008; Shabrawi-Caelen et al., 2004). Most of these articles address the diagnostic pitfalls in cases with VLS. In a case series of 11 subjects with four juveniles (Carlson et al., 2002), the clinicopathological findings for melanocytic nevi occurring in LS, which can mimic malignant melanoma, are described. In one series of 58 pigmented genital lesions in women (Ribé, 2008), there were six patients with atypical genital nevus (AGN) with a mean age of 21 years and six with MVM with a mean age of 55 years; the youngest MVM case aged 23 years. Of all nevi in a pediatric dermatology practice, 3.5% were genital (Hunt et al., 2014).

#### Cohort and epidemiologic studies including cases of nevi or MVM in juveniles

Seven publications were included (Blessing et al., 1991; Cohen Goldemberg et al., 2020; Hieta et al., 2019; Ragnarsson-Olding et al., 1993; Rock et al., 1990; Sanchez et al., 2016; Woolcott et al., 1988). In a prospective study of 301 women seen in general gynecologic practice (Rock et al., 1990), 12% had a pigmented lesion of some kind or hyperpigmentation, whereas 2.3% of the subjects had a vulvar nevus. The one patient aged 19 years in this cohort had the only dysplastic nevus. In a population study with regional data from the United States in 1973‒2010, of 1,463 cases of vulva or vaginal melanoma, 13 cases were aged 10‒19 years. A 10-year survival in the juveniles was 100% (Sanchez et al., 2016). A brief communication based on population data from Finland (Hieta et al., 2019) studied the possible relation between LS and MVM and found a relative risk for MVM of 341 for women with VLS compared with that for women without. The age of the subjects was not given. A national Scottish study (Blessing et al., 1991) found 41 cases of MVM in the period 1979‒1989, with one subject being aged 11 years and all the other cases being aged ≥40 years. Recent data from Brazil (Cohen Goldemberg et al., 2020) found three adolescents with MVM of 801 mucosal melanomas documented in females. Data from Sweden (Ragnarsson-Olding et al., 1993) documenting MVM found 245 cases before 1984 with an average age of 67.7 years, a range of 18‒91 years, and one juvenile.

#### Histopathology and immunohistochemistry of melanoma vulvar nevi

A total of 13 publications on the histopathology and immunohistochemistry of melanomas and melanocytic lesions met the inclusion criteria (Ahn et al., 2016; Blessing, 1999; Brenn, 2011, 2018; Christensen et al., 1987; Clark et al., 1998; Cook, 2010; Elder, 2006; Fu et al., 2021; Haupt and Stern, 1995; Mason et al., 2011; Skelton et al., 1991; Wick, 2015). Clark et al. (1998) introduced the term atypical melanocytic tumor of the genital type, subsequently abbreviated to AGN. An AGN has a relatively specific morphology and may be regarded as belonging to the class of nevi with special-site features. The specific features of AGN comprise characteristics such as symmetry and the presence of borders, which ultimately can only be investigated on a diagnostic excision. Therefore, Brenn (2011) advocates diagnostic excision to adequately differentiate these lesions from melanoma. He also states that in the background of lichen sclerosis, the differentiation of vulvar nevi from melanoma is even more challenging, likely because inflammation can lead to cytological atypia in melanocytes and that epidermal atrophy and clefting can mimic the epidermal consumption sometimes seen in melanoma.

#### Systematic and comprehensive reviews

Three systematic reviews were included. Gadducci et al. (2018) showed that vulvar melanoma is a rare disease in women, with a mean age of onset of 54‒76 years, a very poor prognosis, and a median overall survival of 41 months. This publication includes but does not differentiate for juveniles. From 1970 to 2020, there were 100 case reports of vulvar malignancies up to age 21 years, of which six were melanomas, all with VLS (Strickland and Fadare, 2021). A review on vulvar melanocytic lesions (Murzaku et al., 2014) found the median age of patients with common vulvar nevi to be 28‒33 years, whereas the median age of those with atypical melanocytic tumor of the genital type was 17‒26 years.

In all the literature studied in this scoping review, 42 possible MVM in juveniles were reported with no mortalities (Table 2).

**Table 2:** Publications Including Juveniles with Malignant Vulvar Melanomas

References, Country	Number of Juveniles/Total Number of Subjects (Age, y)	LS Present	Treatment	Mortality Juveniles
**Case report juvenile vulvar melanoma**				
Egan et al., 1997	2 (ages 9 and 11)	Yes	Shaving followed by local excision	No
Filippetti and Pitocco, 2015	1 (age 14)	No	Amelanotic melanoma, SN negative	No
Friedman et al., 1984	1 (age 14)	Yes	Bilateral excision of labia minora and lymph node (nodes negative)	No, NED after 1 year
Hassanein et al., 2004	1 (age 10)	Yes	Partial vulvectomy	No, NED after 1 year
Hulagu and Erez, 1973	1 (age 9)	n.s.	Local excision clitoral melanoma	No, NED after 8 years
La Spina et al., 2016	1 (age 11)	Yes	Regional excision	No, NED after 1 year
Rosamilia et al., 2006; Wechter et al., 2004	1 (age 10)	Yes	Regional excision, lymphadenectomy, adjuvant IFN	No, NED after 2.6 years
Webber et al., 2008	1 (age 12)	n.s.	Local re-excision	No
Spatz et al., 1998	1 (age 28 at diagnosis of malignant blue nevus, lesion present from age 12)	No	Wide local excision, regional node-negative, ovarian metastasis at age 43 y	no, NED 1 year after ovariectomy
**Case series of vulvar melanomas, including one or more juveniles**				
Ariel, 1981	1 of 45 (age 15, case dates from before 1965)	n.s.	n.s	n.s
Chung et al., 1975	1 of 44 (age 17)	n.s.	n.s.	n.s.
De Simone et al., 2008	1 of 10 (age 15)	n.s.	Excision of 2 cm margin	no, NED after 6 years
Eleno Beierbach et al., 2020	1 (1 vulva in a series of 16 pediatric melanoma)	n.s.	n.s.	n.s.
Jaramillo et al., 1985	1 of 16 (probably one juvenile, aged 18)	n.s.	n.s.	n.s.
Morgan et al., 1988	1 of 18 (age 18)	n.s.	Wide local excision	NED after 7 years
Nagarajan et al., 2017	1 or 2 of 100 (age 18)	n.s.	n.s.	n.s.
Räber et al., 1996	1 of 89 (age 18)	n.s.	n.s.	n.s.
Rao et al., 1990	1 (age 8 perineum in a series of 33 pediatric melanoma)	n.s.	Wide local excision, node biopsy	NED after 8 years
Rouzbahman et al., 2015; Sinasac et al., 2019	1 of 50 (age 17)	n.s.	n.s.	n.s.
Trimble et al., 1992	1 of 80 (two cases, one is same case as Chung)	n.s.	n.s.	n.s.
**Cohort and epidemiology including one or more juveniles**				
Blessing et al., 1991, Scotland	1 of /41 (age 11, all other cases aged ≥40)	n.s.	Local excision, superficial	NED after 3 years
Cohen Goldemberg et al., 2020, Brazil	3 of 270 (ages 10‒19)	n.s.	n.s.	n.s.
Hieta et al., 2019, Finland	Age not stated, three MVM in LS population	3 of 9 MVM had LS	n.s.	n.s.
Ragnarsson-Olding et al., 1993, Sweden	1 of 219 probably, one case (age 18)	n.s.	n.s.	n.s.
Sanchez et al., 2016, USA	13 of 1,463 (age 10‒19)	n.s.	n.s.	100% 10-year survival
Woolcott et al., 1988, Australia	≥1 (range = 15‒91); number of juveniles not stated	n.s.	n.s.	n.s.

Abbreviations: LS, lichen sclerosus; MVM, malignant vulvar melanoma; NED, no evidence of disease; n.s., not stated; SN, sentinel node.

Total number of possible juvenile MVM: 42 juveniles included in case report, cohort, or epidemiologic studies. For mortality, no mortality was reported.

### Cohort of nevi with and without a background of JVLS demographics from the Netherlands

**Figure 2:**
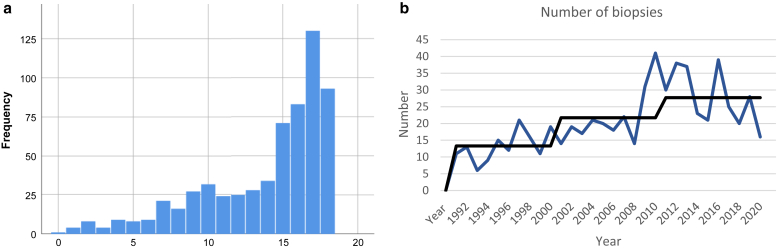
**Biopsies of vulvar melanocytic lesions in juveniles in the Netherlands in 1991‒2020.** (**a**) Age at biopsy. (**b**) Year of biopsy. The number of cases per year is represented by the blue line, and the average per decade is represented in the black line.

From the Pathologische-Anatomisch Landelijk Geautomatiseerd Archief, Dutch Pathology Registry database, 627 cases of females aged ≤18 years in the Netherlands were anonymously identified in 1991‒2020 with a histologically diagnosed vulvar melanocytic lesion. Age is shown in Figure 2a. The number of biopsies with a melanocytic vulvar lesion in juveniles has doubled, from an average of 13.3 per year during 1991‒2000 to an average of 27.7 per year during 2010‒2020 (Figure 2b). No vulvar melanomas in juveniles were reported in the 30 years of the database, independently verified by the Netherlands Comprehensive Cancer Organization, where no malignant melanomas of the female genital tract in patients up to age 18 years through the year 2020 were found. In the histology reports in the Pathologische-Anatomisch Landelijk Geautomatiseerd Archief, Dutch Pathology Registry, atypia or dysplasia was recorded in 29 cases (4.6%), and LS was recorded in 16 cases (2.6%). In six cases with VLS, the pathologist reported atypia or dysplasia (36% of the cases with VLS). Follow-up data of the 627 subjects through October 2021 revealed no melanomas but two nongenital premelanoma lesions. A patient aged 8 years with a vulvar melanocytic nevus in 1994 developed a lentigo maligna of the eyelid at age 32 years. In another case, a patient aged 18 years with a vulvar compound nevus in 1995 was diagnosed with melanoma in situ on the lower leg at age 43 years. The average follow-up was 13.43 years, range of 10 months to 30 years.

### Histopathology of a cohort of juvenile cases of a vulvar nevus with concomitant JVLS

**Figure 3:**
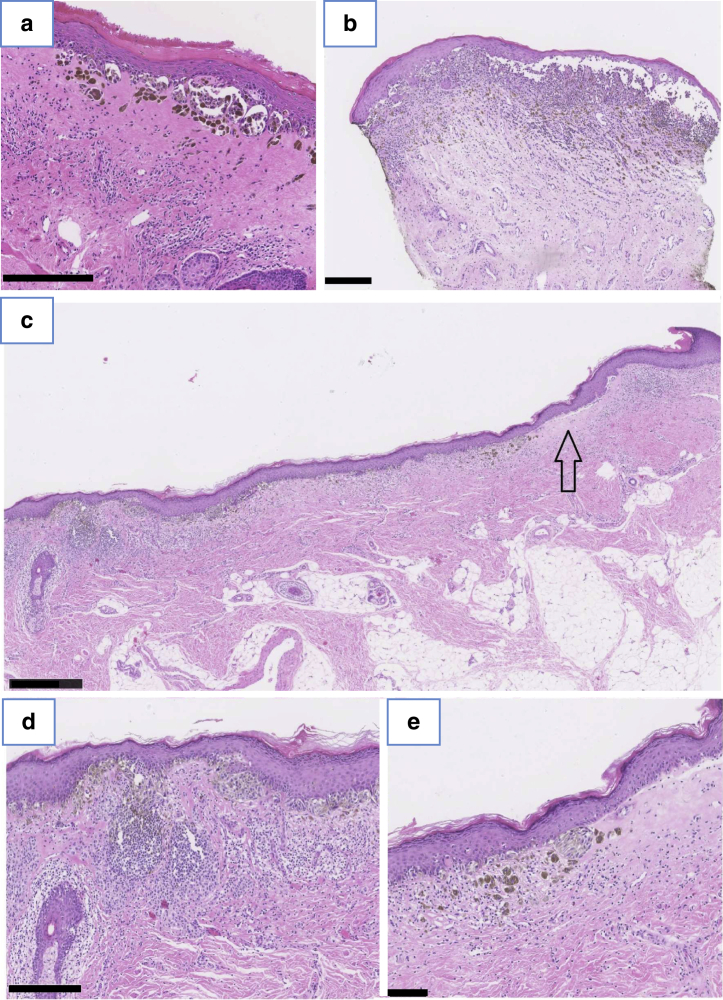
**The histological spectrum of nevi in a background of juvenile vulvar lichen sclerosus.** (**a**) A heavily pigmented junctional nevus in the background of vulvar lichen sclerosus in a patient aged 3 years. (**b**) Compound nevus with dyshesive pattern in the background of vulvar lichen sclerosus. (**c‒e**) A compound vulvar nevus in the background of vulvar lichen sclerosus in a patient aged 10 years. (**c**) Overview (at arrow magnified in **e**). (**d**) Inflammation. (**e**) High-power view (at the arrow in **c**). Bar = 0.250 mm in **a, b,** and **d;** 0.500 mm in **c**; and 0.100 mm in **e**.

**Figure 4:**
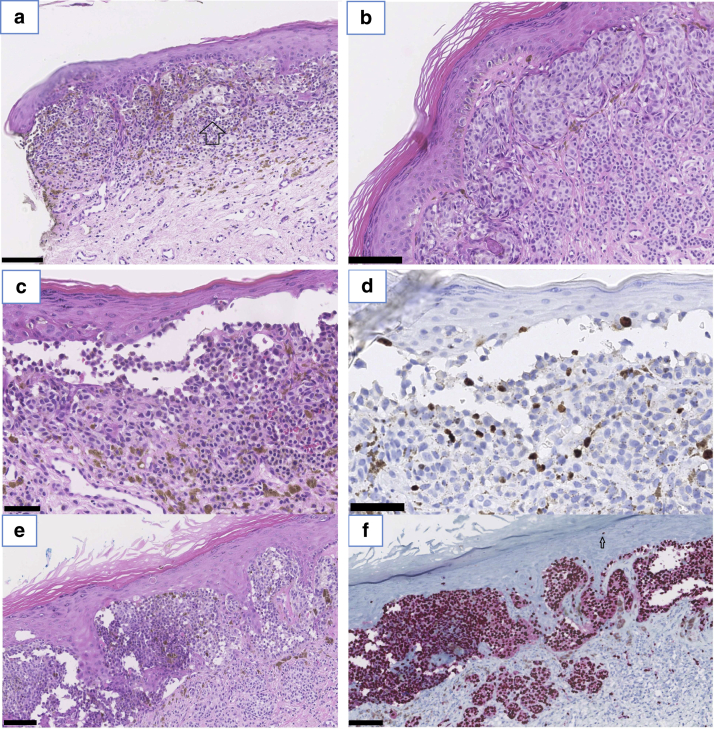
**Atypical features seen in vulvar nevi in a background of juvenile vulvar lichen sclerosus.** (**a**) A compound nevus in a patient aged 11 years, at arrow cells with a relative increase in the cytoplasm. (**b‒f**) A compound vulvar nevus in a patient aged 14 years. (**b**) Cytonuclear atypia with dermal maturation. (**c**) High-power H&E stain. (**d**) Ki-67 positivity in the same location as in **c**. (**e**) High-power H&E stain. (**f**) SOX10 expression in the same location as in **e** showing the distribution of melanocytes. Bar = 0.100 mm in **a, b, e**, and **f** and 0.05 mm in **c** and **d**.

Of the total 627 juvenile vulvar nevi, the records of 16 cases reported concomitant JVLS, tissue samples were available from 12 of these 16 cases (75%) of vulvar melanocytic lesions with concomitant JVLS in the Netherlands in 1991‒2020. Five biopsies of vulvar nevi in juveniles without JVLS were retrieved from our own archive for comparison. Cases with and without VLS were compared (Table 3). Moderate to severe cytological atypia was seen in 83% of the cases with VLS and 40% of the cases without. In cases with VLS, architectural atypia was seen in 83% versus that seen in 40% without VLS. Focal pagetoid spread was found in about 40% regardless of the presence of VLS. A slightly increased proliferation fraction was only seen in the Ki-67 staining when VLS was present. Furthermore, the dyshesive pattern was more often seen in patients with LS. Features of LS such as inflammation and homogenized stroma were logically more often seen in VLS cases. PRAME was always negative. These characteristics are illustrated in Figures 3 and 4.

**Table 3:** Scoring Vulvar Melanocytic Nevi in Juveniles with and Without Lichen Sclerosus

Stain	Characteristic	Grading	Results with LS	Results without LS
	Age range, y		3‒16	5‒17
	Number of cases		12	5
**H&E**				
	Most prominent pattern, if present	None/nested/crowded/dyshesive	Dyshesive (67%)	Nested, 4 (80%)
	Type of nevus	Intradermal/junctional/compound	Compound (58%)	Compound, 5 (100%)
	Junctional component: nested pattern	No/yes/not applicable	4 (33%)	3 (60%)
	Junctional component: dyshesive pattern	No/yes/not applicable	10 (83%)	0 (0%)
	Junctional component: crowded pattern	No/yes/not applicable	1 (8%)	0 (0%)
	Cell nests bulge downward from tip rete ridges	No/yes/not applicable	3 (25%)	2 (40%)
	Splitting dermal epidermal junction	No/yes/not applicable	11 (92%)	0 (0%)
	Cytological atypia	No/mild/moderate/severe	Moderate or severe, 10 (83%)	Moderate or severe, 2 (40%)
	Architectural atypia	No/mild/moderate/severe	Mild or moderate, 10 (83%)	Mild or moderate, 2 (40%)
	Homogenized dermis	No/homogenized/dense	11 (92%)	0 (0%)
	Melanocytes in fibrotic papillary dermis	No/yes/not applicable	7 (58%)	0 (0%)
	Dermal maturation	No/yes/not applicable	7 (58%)	5 (100%)
	Mitotic activity intraepidermal	No/yes/not applicable	2 (17%)	0 (0%)
	Mitotic activity intradermal superficial	No/yes/not applicable	0 (0%)	0 (0%)
	Mitotic activity intradermal deep	No/yes/not applicable	0 (0%)	0 (0%)
	Ulceration	No/yes	0 (0%)	0 (0%)
	Lymphocytic infiltration present	No/yes	11 (92%)	0 (0%)
	Heavily pigmented	No/yes	12 (100%)	2 (40%)
	Adnexal involvement	No/yes	0%	1 (20%)
	Multinuclear cells present	No/yes	1 (8%)	0 (0%)
**Immunohistochemistry**				
SOX-10	Ascending	No/sparsely ascending/ascending	5 (42%)	2 (40%)
	Pagetoid upward spread nests or cells	No/focal/extensive	5 (42%)	2 (40%)
HMB-45	Intraepidermal	No/gradient/strong	Strong, 12 (100%)	Strong, 4 (80%)
	intradermal	No/gradient/strong/not applicable	7 (58%)	5 (100%)
Ki-67		<1, 1‒10, and >10%	11 (92%) Ki-67 low-grade positive	0 (0%)
PRAME		Negative/focal/170%/>70% positive	0 (0%)	0 (0%)

Abbreviation: LS, lichen sclerosus.

## Discussion

This scoping review shows that juvenile vulvar melanoma is extremely rare, and this is substantiated by the population data from the Netherlands. We confirm with a large case series that in a background of juvenile VLS, histopathological analysis can show atypical features that could raise suspicion of melanoma despite having a benign course. Thus, we conclude that (diagnostic) excision biopsies in this age group are not indicated even when atypical histological features are observed.

It may be questioned whether an MVM in a juvenile exists at all. The literature reveals a minimal number of cases, and even these cases have led to polemics regarding the correct diagnosis. Only one lymph node metastasis was ever reported (Wechter et al., 2004), whereas only a distant metastasis is regarded as the definitive proof of malignancy in melanocytic tumors (Mooi, 2014). One case of the extremely rare malignant blue nevus in an adult, probably stemming from a vulvar blue nevus observed in adolescence, is reported to have led to an ovarian metastasis 31 years after the nevus was first seen (Spatz et al., 1998). It would be instructive to review the histology of a number of these cases with a panel of experts.

Cohort studies and epidemiologic studies of MVM that include juveniles are nearly all studies from before the year 2000 and are always with a favorable outcome for juveniles (Table 2) compared with the vast majority of MVM that show a poor prognosis. The question arises regarding the possibility that these earlier documented lesions were misdiagnosed or that these melanomas in juveniles have such a favorable prognosis compared with adult vulvar melanoma and conventional cutaneous melanoma that the term melanocytoma (low-grade melanocytic neoplasm) (Elder et al., 2020; Schaffer and Orlow, 2005) would be more appropriate. Vulvar melanoma in a juvenile has not been seen in 30 years in the Netherlands, nor did any melanomas develop after a vulvar nevus in a juvenile. It is highly unlikely that the two premalignant lesions reported 24 and 25 years after a vulvar melanocytic lesion were related to the original nevus, considering their locations (eyelid and lower leg). Thus, our scoping review and population study together give credibility to questioning the existence of MVM in juveniles.

Genital nevi in a background of LS are regarded as more challenging to differentiate from MVM (Brenn, 2011). AGN lesions are more likely found in younger subjects (Brenn, 2018; Ribé, 2008). Considering the unlikelihood that a lesion is an MVM in a juvenile, we question the advice (Brenn, 2011, 2018) to excise the lesion in juvenile cases. Nevertheless, our study shows a doubling of biopsies in the last 30 years in the Netherlands, likely reflecting defensive medicine (Welch et al., 2021).

Despite a worrisome histological pattern that is seen in many cases of vulvar melanocytic lesions in juveniles, subsequent melanomas did not develop. In the tissue samples we examined, comprising three quarters of all cases of vulvar nevi with JVLS in the Netherlands over the past 30 years, we mainly found cytological and architectural atypia, in combination with a slightly higher proliferation fraction. A dyshesive pattern and inflammation were often seen, which in the absence of VLS are atypical features raising suspicion of melanoma (inflammation mostly in mucosal melanoma) (Busam et al., 2019). This dyshesive pattern is described (Clark et al., 1998) in a subset of AGN. In contrast to the description of AGN, vulvar melanocytic lesions in the background of VLS are usually without distinct borders and have lentiginous growth (Brenn, 2011).

Kurman et al. (2019) elucidates the differences between atypical vulvar nevi and dysplastic vulvar nevi. Dysplasia implies architectural and cytological atypia but with some additional features. “Characteristics that help to distinguish AGN from melanoma include the presence of dermal maturation, the sparsity of mitotic activity, and the absence of necrosis or ulceration” (Kurman et al. [2019] citing Murzaku et al. [2014]). The use of the term dysplastic nevus has varied over the years. Furthermore, some interpret dysplasia as a premalignant condition, which is not the case in the series of biopsies we examined. In our opinion, these vulvar nevi in the context of VLS are a distinct subset of nevi that show atypical features mainly owing to the inflammatory reaction and are not indicative of premalignancy. Thus, using the term dysplasia in this context has no additional value above the combination architectural and cytological atypia, and the term dysplastic should be avoided in this context.

In our series, at a median follow-up of 13 years, no melanoma or metastasis occurred. This supports a policy of utmost restraint, confirming that there is little or no necessity for a diagnostic biopsy or excision of a vulvar melanocytic lesion in a juvenile. If biopsied, a diagnosis of melanoma should be avoided because these lesions do not show distant metastasis when found in juveniles (Mooi, 2014).

The strength of this study lies in the systematic and comprehensive evaluation of the literature combined with a large population study in the Netherlands and verifying the population data with a second independent national database.

Limitations include the fact that owing to anonymity, there is no clinical information. In addition, a limitation is that the literature is, for the most part, descriptive. Neither were we able to elucidate the relationship of LS with the atypical histopathological features observed, one of the questions motivating this study. Future guidelines should differentiate for age and anatomical location, with a high threshold of suspicion before biopsy of vulvar lesions in juveniles and a low threshold for referral, with advice for having a consultation in a center of expertise.

Previous studies on vulvar nevi or vulvar melanoma in juveniles have generally been anecdotal. This scoping review shows just how unlikely a vulvar nevus in a juvenile is a melanoma. The population data show that most vulvar nevi in a background of VLS in juveniles had atypical features with no subsequent metastasis. Clinicians should be extremely hesitant to excise a vulvar nevus in a juvenile, and pathologists should beware of overdiagnosis when interpreting findings in a biopsy, especially in a setting of LS. Clinicians and pathologists must work together so that clinical decision making is well-founded when a vulvar melanocytic lesion with or without VLS in a juvenile shows signs of atypia. Our findings reflect the need for specific guidelines for care of juveniles with vulvar melanocytic lesions, and considering the rarity of such lesions and the consequences of potentially unnecessary diagnostics or treatment, we recommend referral to a center where knowledge can be maximized.

## Materials and Methods

### Scoping review of the literature

A scoping review of the literature was performed systematically according to the preferred reporting items for systematic reviews and meta-analysis guidelines (Tricco et al., 2018).

All published literature on the clinics and histopathology of vulvar nevi with or without VLS or vulvar melanoma in juveniles up to September 2021 were studied. The search strategy was developed encompassing five databases: Embase, Medline ALL, Web of Science Core Collection, Cochrane Central Register of Controlled Trials, and Google Scholar 100 top ranked. Screening for relevance based on title and abstract was done by one reviewer (BM), uncertainties were discussed with other authors (ALM, IAMvdA), and if necessary, the material and methods and results sections of the publication were perused to ensure that no case of juvenile vulvar melanoma was overlooked. Final eligibility was based on full-text reading (BM) and consultation when needed (ALM, IAMvdA). References in the included publications were checked for relevant publications. Included articles were grouped and summarized according to their focus: case report and series, epidemiology, histology, immunohistochemistry, and systematic review.

### Cohort of nevi with and without a background of JVLS demographics from the Netherlands

A search was performed through the national cytohistopathology database of the Netherlands, Pathologische-Anatomisch Landelijk Geautomatiseerd Archief, Dutch Pathology Registry, for biopsy reports on females aged ≤18 years in the period 1991‒2020, including anatomical locations of vulva, labium, clitoris, or perineum in which a melanocytic lesion of any kind was recorded. In addition, all follow-up histology reports of these subjects were retrieved. Descriptive statistics regarding demographics and histological findings were calculated using the Statistical Package for the Social Sciences 25.

An independent query was submitted to the National Cancer Registry of the Netherlands (Netherlands Comprehensive Cancer Organization) for any melanoma of the genital tract in females aged up to age 18 years.

### Histopathology and immunohistochemical analysis of a series of melanocytic lesions with and without a background of JVLS

The vast majority of publications on the histopathology of nevi of special sites, including vulvar nevi, and MVM refer to a few seminal publications (Brenn, 2011; Clark et al., 1998; Elder, 2006) when discussing special-site nevi of the vulva. Using the characteristics described in these publications as well as the World Health Organization publication (Elder et al., 2020) and textbooks (Busam et al., 2019; Kurman et al., 2019), the data were analyzed in a standardized and semiquantitative manner. The stains H&E as well as SOX10 (clone SP267, Cell Marque, Rocklin, CA), HMB-45 (Ventana Medical System, Oro Valley, AZ) (Skelton et al., 1991), and Ki-67 (clone 30-9, Ventana Medical System) (Carlson et al., 2002; La Spina et al., 2016; Pinto et al., 2012) (markers for melanocytic lesions and proliferation) and PRAME (clone EPR20330, Abcam, Cambridge, United Kingdom) (Lezcano et al., 2018) (a marker that is preferentially expressed in melanoma) were applied. From a previous study, (Morrel et al., 2020), all cases of nevi in a background of JVLS in the Netherlands were identified, and material, if available, was obtained. In addition, we identified and retrieved material from all cases of vulva nevi in juveniles from a single center. Scoring was done by two dermatopathologists (ALM, JD) using the list of predefined histopathological features based on the literature.

### Data availability statement

Raw data were generated through the Pathologische-Anatomisch Landelijk Geautomatiseerd Archief, Pathology Registry of The Netherlands, queries numbers LZV2020_195A1 and LZV2020_195A2, and at repository EMCD18041 of the Department of Dermatology, Erasmus MC University Medical Center (project identification number 3608-Oracle 7402). The data that support the findings of this study are available from the corresponding author (ALM) on reasonable request.

## ORCIDs

Beth Morrel: http://orcid.org/0000-0001-7609-7521

Irene A.M. van der Avoort: http://orcid.org/0000-0003-1467-6290

Jeffrey Damman: http://orcid.org/0000-0001-5997-7551

Antien L. Mooyaart: http://orcid.org/0000-0001-9810-5780

Suzanne G.M.A. Pasmans: http://orcid.org/0000-0003-1018-4475

## Author Contributions

Conceptualization: BM, IAMvdA, JD, ALM, SGMAP; Data Curation: BM, IAMvdA; Formal Analysis: BM; Investigation: BM, IAMvdA, JD, ALM, SGMAP; Methodology: BM, IAMvdA, JD, ALM, SGMAP; Supervision IAMvdA, ALM, SGMAP; Visualization: BM, IAMvdA, JD, ALM, SGMAP; Writing - Original Draft Preparation: BM, IAMvdA, ALM, SGMAP; Writing - Review and Editing: BM, IAMvdA, JD, ALM, SGMAP

## Conflict of Interest

The authors state no conflicts of interest.
